# Group Reunion in Zoo European Wildcats Using Cat Appeasing Pheromone (CAP) and Gradual Release of the Animals in the Exhibit—A Case Study

**DOI:** 10.3390/ani12101302

**Published:** 2022-05-19

**Authors:** Valentina Bertoni, Caterina Spiezio, Barbara Regaiolli, Alessandro Cozzi, Paola Valsecchi, Simona Normando

**Affiliations:** 1 Research & Conservation Department, Parco Natura Viva—Garda Zoological Park, 37012 Bussolengo, Italy; valentinabertoni4@gmail.com; 2 Research Institute in Semiochemistry and Applied Ethology, 84400 Saint-Saturnin-lès-Apt, France; a.cozzi@group-irsea.com; 3 Department of Chemistry, Life Sciences and Environmental Sustainabilty, University of Parma, 43124 Parma, Italy; paolamaria.valsecchi@unipr.it; 4 Department of Comparative Biomedicine and Food Science, University of Padua, 35020 Padua, Italy; simona.normando@unipd.it

**Keywords:** wildcats, CAP semiochemicals, social dynamics, group reunion

## Abstract

**Simple Summary:**

The present paper is, to our knowledge, the first study that documents the behavioural responses of a group of wildcats kept in a controlled environment upon reunion with one previous member of the social group, a female wildcat, after a long time of separation. The reunion process was gradual, and CAP pheromones were used to minimise agonistic behaviour and to increase the probability of the acceptance of the female. In the first days after the reunion, there was an increase in species-specific behaviours and a reduction in aggressive interactions, but the aggression threshold appeared to lower again after one month, with the behavioural patterns of the late-reunion periods resembling those of the pre-reunion period.

**Abstract:**

The union or reunion of animals with social groups can be a challenging situation, and little has been published about it when solitary species are concerned. Therefore, the aim of the present study was two-fold: (1) to advocate the need for systematic publications about strategies and the outcomes of reunion episodes in zoos and other facilities; and (2) to describe the behaviour of European wildcats (*Felis silvestris silvestris*) during one such episode, in which a female cat was reintroduced into her family social group using a gradual reunion procedure and cat appeasing pheromone (CAP) (spot-on). The study comprised three periods: the pre-reunion period (10 days, 20 sessions per wildcat), the post-reunion period (A, 5 days, 10 sessions per wildcat; B, 5 days, 10 sessions per wildcat) and the late-reunion period (5 days, 10 sessions per wildcat). In the post-reunion periods, all wildcats were together in the enclosure and were spotted with CAP pheromone. Per period, we collected data on individual and social behaviours. Individual behaviours, such as attention and self-grooming, were performed more in the pre-reunion than in the post-reunion period. Regarding social behaviours, we found that agonistic behaviours were performed more in the pre-reunion than in the post-reunion period. We observed behavioural changes over the course of the study, with behavioural patterns of the late-reunion periods resembling those of the pre-reunion period.

## 1. Introduction

The reunion/union of new individuals with pre-existing social groups can be a challenging situation for most animal species; this can also be the case when attempting the reunion of an animal with his/her previous social group after long-term separation due, for example, to veterinary practices [1,2,3,4,5]. Indeed, conflicts among animals kept in social groups can be problematic and require careful management in controlled environments, as they can have relevant welfare consequences. Agonistic behaviours, though part of the species-specific behavioural repertoire of a species, can have negative and even fatal consequences for the animals involved, especially in controlled conditions, and their increase could be linked to a decrease in animal welfare level [6,7,8]. Deciding whether and how to proceed with a (re)union can be difficult, and evidence-based research can play an important role in such decisions.

Evidence-based medicine/practice is a conceptual model that aims to provide “the right care at the right time to the right individual” using a multidisciplinary approach, which conscientiously, explicitly and thoughtfully integrates research evidence, professional expertise and patient’s related variables [9]. An evidence-based practice approach could fit all the requirements to be beneficial when facing difficult decisions, such as the ones regarding (re)union of animals into pre-existing social groups. However, at present, someone wishing to use such an approach when considering a (re)union would be virtually unable to do so, for several reasons. One reason is that, even if there were many published papers on the topic, they would be unlikely to be high in the evidence pyramid, such as randomised blind controlled studies, because in such situations, there is usually no control group (i.e., one specific animal should be introduced into one specific group), and blindness is very difficult to obtain. Moreover, (re)unions occur or can occur but are not very frequent events in zoos; therefore, the zoo professionals making the decision are unlikely to have considerable past experience of (re)union episodes in that species, also given the variety of species housed in zoological parks. Individuals can differ widely in their temperament, age, past history and reactivity [10]. Animals in a controlled environment are managed in ways that are likely to differ in some relevant characteristics from what would occur in their original environment, in terms of adaptation. For example, for many species, the space available in a controlled environment is significantly less than the territory/home range they would have in the wild, but their need in terms of trophic resources is met independently of the availability of a big enough territory/home range that ensures them enough food. A similar consideration can be proposed for species that have evolved a solitary lifestyle supposedly due to prey/food availability constraints. Further investigation is needed to understand whether an individual would benefit from pair/group living in a controlled environment with a relatively limited space or whether they would still need to roam alone [11]. Although most of the abovementioned limitations are unlikely to be eliminated, to improve the situation, it is important for professionals to consistently publish all their experiences and strategies concerning (re)union episodes (whatever the outcome), even in the form of case studies. In the greater long term, this might enable the performance of a meta-analysis, which could provide some scientifically based guidance on whether and how to (re)introduce animals. Meanwhile, sharing such experiences could help professionals make better decisions regarding possible (re)unions at their facility to minimise risks to the welfare of the animals involved or even fatal outcomes. At the moment, the low number of published papers dealing with (re)unions is unlikely to represent a high percentage of the actual cases of (re)unions taking place in zoological institutions worldwide. Moreover, published papers tend to deal mainly with highly charismatic social species (such as social primates, wild dogs and elephants [3,12,13,14,15]), although solitary species can also be kept in groups or pairs in zoos, and such an approach has been found to be beneficial for at least some of them [8,16,17,18,19]. For example, a quick search on Scopus (https://www.scopus.com/; accessed on 16 November 2021) with the introduction AND zoo AND cat AND social AND group as keywords rendered only one result: a paper on enrichment in tigers, including a general section on social housing [20], although two cases of a semiochemical being used during the introduction of tigers have been published elsewhere [2,21].

The addition of semiochemicals is among the strategies used during the formation of social groups or the union of individuals with a pre-existing group to improve the chance of success [2,5]. Semiochemicals are chemical compounds involved in the interaction between organisms [22,23], and pheromones are a subclass of semiochemicals involved in the communication between individuals of the same species. Pheromones elicit a specific behavioural and/or physiological response in animals able to perceive them [22,23,24,25]. Nevertheless, the response of an individual can be modulated by age, sex, hormonal state, genetics, experience of the recipient and other context-specific variables [23,24]. Cats have well-developed chemosensory gene families, especially coding for vomeronasal receptors, and rely extensively on pheromones for social communication [26].

In a controlled environment, pheromones are mainly used as sensory environmental enrichment [27,28] to decrease stress [29], to promote positive social relationships [30,31] and to decrease abnormal behaviour [32]. In felids, the pheromones used are derived from the domestic cat (*Felis catus*) and include two facial semiochemicals, Fraction 3 of facial pheromone (F3) and Fraction 4 of facial pheromone (F4), and a maternal pheromone, the Cat Appeasing Pheromone (CAP) [33]. Facial semiochemicals are secreted by sebaceous glands of the cat’s cheeks and are involved in territorial marking, as well as in complex social interactions. In domestic cats, facial semiochemicals play an appeasing role and can facilitate social bonding and relationships [25]. The CAP is secreted by sebaceous glands of the mammary sulcus by the mother cat a few days after parturition [25], and, in recent years, its synthetic analogue (a mixture of myristic, lauric, palmitic, linoleic, oleic and stearic acids [34]) has been produced and tested on domestic cats, causing a decrease in antagonistic behaviour [35,36]. In recent years, several studies have investigated the effect of the domestic cat’s semiochemicals on the behaviour of other species of felids in zoos to improve their welfare and/or to help manage their social dynamics [2,17,29,30,31,32,35] (see Ref. [17] for the absence of an effect in snow leopards). However, to our knowledge, no study has described the use of domestic cats’ pheromones on the behaviour of the European wildcat (*Felis silvestris silvestris*).

The European wildcat is one of the five existing subspecies of wildcats [37]. It is considered threatened and is strictly protected under Annex IV of the European Habitat Directive (92/43/EEC) [38,39]. The population of European wildcats is decreasing, and interbreeding with feral domestic cats represents a threat to the conservation of the wild population’s genetic pool. In the perspective of its conservation and reintroduction in areas where it is now extinct (i.e., England), ex situ reproduction is needed. European wildcats are solitary and territorial. Males and females meet each other only in the mating period; after that, the female gives birth to her cubs and stays with them until they become independent—at approximately five months of age—and then, the kittens disperse. Wildcats reach sexual maturity when they are about five years old [38,40,41,42].

The aim of the present study is two-fold:

1. To advocate the need for systematic publications on the strategies enacted and on the outcomes, both in the short and long term, of as many (re)union episodes as possible in zoos and other facilities housing animals worldwide.
2. To describe the behaviour of a family of zoo European wildcats during one such episode, involving a procedure aimed at the reunion of a female cat with her family social group after seven months of absence due to medical reasons.

To compensate for the fact that housing European wildcats in groups under human care can represent an unnatural situation for this species and to improve the likelihood of success, the procedure included the use of CAP on all the animals and was performed gradually through the reunion of one cat at a time, until the separated female was in the enclosure with all conspecifics. The choice to include CAP administration in the group reunion procedure was based on the fact that European wildcats and domestic cats have fewer behavioural differences than expected [43] and that different big cat species have been found to respond positively to domestic cat semiochemicals [2,17,29,31,32]. 

## 2. Materials and Methods

### 2.1. Subjects and Area

This study was carried out from June to September 2017. The family group hosted at Parco Natura Viva, Garda Zoological Park (Italy), consisted of five European wildcats: a mother, born in 2007, with her offspring (two males and two females), born in 2012. The wildcats were in the same enclosure before and during the study period. One of the daughters (hereafter, the related female RF) presented health problems and an injury on the back; thus, she needed to be separated from the group and kept in a different enclosure from January to July 2017 for veterinary care and practice. After RF was deemed completely recovered and healthy, the professionals responsible for the welfare of the animals at the zoo had to decide whether to reintroduce her into her natal group. After careful considerations, we planned a reunion procedure based on the following: (1) the history of the cats, as previous research on this same group showed that they displayed a fair amount of affiliation and a very low frequency of agonistic behaviours [43]; (2) the unavailability of another zoo that could accept the RF; (3) the fact that the wildcat enclosure would have granted a better welfare than the one in which the RF would have been hosted if not reintroduced.

To facilitate the reunion of RF with her original social group, a prototype of the CAP pheromone (patent reference: 34) was used on all studied wildcats [2,31]. The CAP was contained in spot-on syringes (2% active compounds), and prototype preparation was carried out by the Department of Chemistry at the “Research institute in semiochemistry and applied ethology (IRSEA)”, Quartier Salignan, France. Before the reunion of the female with the group, the product was applied to all individuals directly on the skin, between the shoulder blades. To do this, the four wildcats were caught using a hand net and immobilised by the keeper while the veterinarian placed the CAP spot-on between the shoulder blades. After that, the four wildcats were moved into an area next to the enclosure until their release.

The wildcats’ enclosure measured 64 m^2^ and contained plants, logs with nest cavities and small wooden houses. The wildcats were fed once a day with one fasting day per week, and water was provided ad libitum. These animals were provided with an environmental enrichment program consisting of different types of manipulative (i.e., paper cups, toys and clothes), sensory (i.e., spices, herbivore faeces, parfums, such as essential oils and scented water) or feeding enrichments (blood ice cube, croquettes and small pieces of meat hidden in manipulative devices) to enhance the performance of natural behaviours.

### 2.2. Preamble

The reunion of wildcats was a gradual process in which all individuals were released back into the original enclosure. In detail, after all cats had been removed from their enclosure to apply the CAP spot-on (17 September), the related female (RF) was first released alone in the enclosure for one hour to allow her to explore the environment. The mother was then released in the enclosure on the same day, whereas the other three wildcats were gradually released during the following day (18 September). The reunion process lasted two days, and no behavioural data were collected during this period, as the data collection started in the pre-reunion period and continued in the post-reunion periods. The cats were periodically, although informally, checked and observed during the day to monitor the situation.

### 2.3. Procedure

Observations of individual and social behaviours were collected before and after the reunion procedure. In particular, data were collected across three different periods: (1) the pre-reunion period without the related female RF; (2) the post-reunion period with RF; (3) the late-reunion period (see Table 1 for a detailed procedure timetable).

Pre-reunion period: This phase lasted 10 days, during which only the mother, the two males and one female were present in the enclosure (RF was still separated from the group). Two 20 min daily sessions were run per subject, one in the morning (9.30 a.m.–12.30 p.m.) and one in the afternoon (2.30 p.m.–5.30 p.m.), for a total of 80 sessions (1600 min).

Post-reunion period: This phase lasted 10 days (divided into post-reunion period A, including the first five days of observation, and post-reunion period B, including the last five days of observation) and was performed after the reunion process was completed, and the five wildcats were together in their home enclosure with RF in the group. Data collection of the group reunion process started two days after the reunion of cats with the related female to complete the formation of the group and to allow the CAP semiochemical to spread through the skin of the wildcats and become effective. Two 20 min daily sessions were run per subject, one in the morning (9.30 a.m.–12.30 p.m.) and one in the afternoon (2.30 p.m.–5.30 p.m.), for a total of 100 sessions (2000 min).

Late-reunion period: This phase lasted five days, and all five wildcats were observed (RF included). Two 20 min daily sessions were run per subject, one in the morning (9.30 a.m.–12.30 p.m.) and one in the afternoon (2.30 p.m.–5.30 p.m.), for a total of 50 sessions (1000 min) of observation. The late-reunion period was compared with the post-reunion periods A and B, as both lasted five days.

According to the aims of this paper, a brief informal update spanning the time from the end of the late-reunion period (15 September 2017) to the present (7 December 2021) will also be given to provide the maximum information possible for a possible future meta-analysis.

### 2.4. Behavioural Observations

The ethogram was defined by direct observations and according to the ethograms described in the literature (Table 2) [44]. The behavioural categories of the ethogram are mutually exclusive. A continuous focal animal sampling method was used to collect durations of individual and social behaviours [45]. The same observer (V.B.) collected data over all the study periods. Individuals were recognised through physical characteristics.

### 2.5. Data Analysis

Two comparisons between/among periods were made:

1. Between the pre-reunion period and the post-reunion period. In this analysis, only data pertaining to the four wildcats who had been in the enclosure in both periods were included (*n* = 4, RF was excluded).
2. Among post-reunion period A, post-reunion period B and the late-reunion period. The data pertaining to all five cats were included in this analysis (*n* = 5, RF was included).

A single-case design was performed to analyse behavioural data of wildcats, using the total duration of each session calculated for each subject as dataset [46,47]. Nonparametric statistical tests were used to compare behavioural data of wildcats between and within periods. The significance level was *p* < 0.05 [48]. In particular, the Wilcoxon test was used to verify the effect of the reunion process on the behaviour of the study wildcats, comparing the pre-reunion and the post-reunion period. Friedman test with Nemenyi post hoc test was used to investigate the effect of the reunion procedure over time by comparing post-reunion period A, post-reunion period B and the late-reunion period with each other. We chose the Nemenyi test as it “was developed to account for a family-wise error and is already a conservative test” [49,50]. The median and interquartile range (IQR) in the Results section are expressed in second(s).

A short descriptive update from the end of the late-reunion period (15 September 2017) to the present was also included, as per the aim of the study.

## 3. Results

### 3.1. Pre-Reunion Period vs. Post-Reunion Period

First, we compared the pre-reunion period with the post-reunion period. In the pre-reunion period, the mother with two males and one female were present in the enclosure, while in the post-reunion period, the related female had been reintroduced into the family group, and the CAP semiochemical had been applied to all wildcats.

#### 3.1.1. Individual Behaviours, Inactivity and “Not Observed”

Medians (IQR) and statistical values of individual behaviours, as well as individual inactivity and “not observed”, are reported in Table 3. Wilcoxon test revealed that among individual behaviours, attention and self-grooming were performed significantly more in the pre-reunion than in the post-reunion period, whereas the opposite pattern was found for “not observed”, which was shown more in the post-reunion period than in the pre-reunion period. No other significant differences were found within individual behaviours, as well as for individual inactivity (see Table 3 for medians, IQR and statistical values). Social inactivity was not performed by any of the four wildcats in either the pre-reunion or the post-reunion period.

#### 3.1.2. Intraspecific and Interspecific Social Behaviour

**Figure 1:**
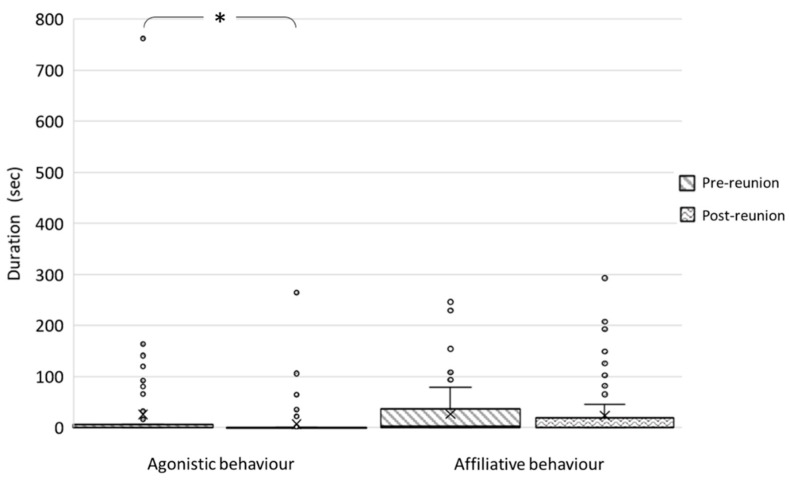
Box and whisker plots of the duration of agonistic and affiliative behaviours of wildcats during the pre-reunion and the post-reunion periods. The horizontal lines within the box indicate the medians, boundaries of the box indicate the 25th and 75th percentile, and the whiskers indicate the minimum and maximum values of the data samples. Outliers are drawn as points. Asterisks indicate a significant difference between periods (*p* < 0.05).

Regarding intraspecific social behaviours, the median (IQR) of agonistic behaviours was 0 s (5.5 s) in the pre-reunion and 0 s (0 s) in the post-reunion period. Wilcoxon test showed that agonistic behaviours were shown significantly more in the pre-reunion than in the post-reunion period (*V* = 2.723, *p* = 0.006) (Figure 1). The median (IQR) of affiliative behaviours was 2.5 s (36 s) in the pre-reunion and 0 s (19.5 s) in the post-reunion period, with no significant difference emerging between the two periods (*V* = 0.819; *p* = 0.413) (Figure 1).

In the case of interspecific social behaviours, the median (IQR) was 4.5 s (34.75 s) in the pre-reunion and 0 s (18.25 s) in the post-reunion period, but no significant differences emerged between the two periods (*V* = 1.219, *p* = 0.223).

### 3.2. Post-Reunion Period A vs. Post-Reunion Period B vs. Late-Reunion Period

We also compared data collected in post-reunion periods A and B and in the late-reunion period.

#### 3.2.1. Individual Behaviours, Inactivity and “Not Observed”

Medians (IQR) and statistical values of individual behaviours, as well as inactivity and “not observed”, are reported in Table 4.

Regarding individual behaviours, significant differences between the periods were found for attention, maintenance, exploration and locomotion (Friedman test: *Q* = 15.891, *p* = 0.0004; *Q* = 6.309, *p* = 0.0427; *Q* = 15.617, *p* < 0.0004; *Q* = 7.924, *p* = 0.0190, respectively), as well as for individual inactivity and social inactivity (Friedman test *Q* = 6.633, *p* = 0.0362; *Q* = 8.857; *p* = 0.0119, respectively). No significant differences were found for self-grooming, territorial behaviours and “not observed” (Friedman test *Q* = 3.500; *p* = 0.1738; *Q* = 0.583, *p* = 0.7470; *Q* = 3.847, *p* = 0.1461).

Nemenyi test revealed that attention was observed significantly more in the late-reunion period than in the post-reunion period A, whereas exploration was performed significantly more in the post-reunion period B and in the late-reunion period than in the post-reunion period A. No other significant differences were reported (see Table 4 for medians, IQR and statistical values).

#### 3.2.2. Intraspecific and Interspecific Social Behaviour

**Figure 2:**
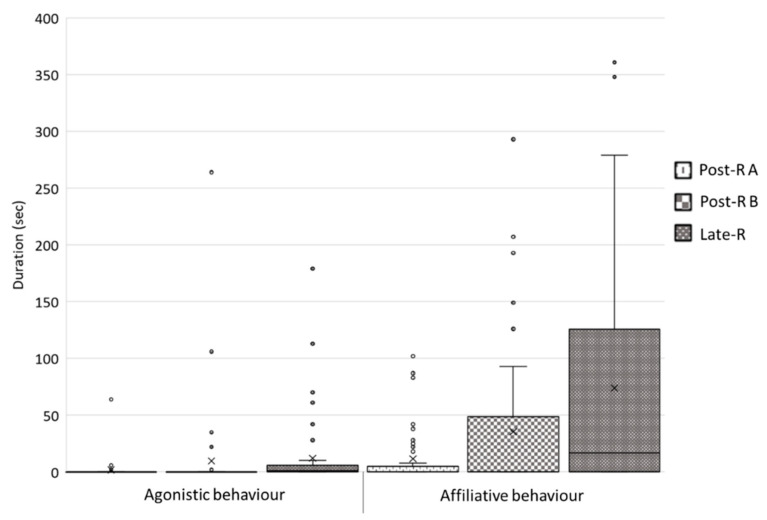
Box and whisker plot of the duration of agonistic and affiliative behaviours shown by wildcats during post-reunion period A (Post-R A), post-reunion period B (Post-R B) and the late-reunion period (Late-R). The horizontal lines within the box indicate the medians, boundaries of the box indicate the 25th and 75th percentile, and the whiskers indicate the minimum and maximum values of the data samples. Outliers are drawn as points.

Regarding intraspecific social behaviours, the median (IQR) of agonistic behaviours was 0 s (0 s) in both post-reunion periods A and B and 1 s (6 s) in the late-reunion period. The median (IQR) of affiliative behaviours was 0 s (5 s) in the post-reunion period A, 0 s (48.5 s) in the post-reunion period B and 17 s (126 s) in the late-reunion period (Figure 2). The two male wildcats performed more agonistic behaviours than other individuals, and most of the aggressive interactions occurred between them.

In the case of interspecific social behaviours, the median (IQR) was 0 s (2.3 s) in the post-reunion period A, 4 s (21 s) in the post-reunion period B and 29 s (54.3 s) in the late-reunion period. Friedman test showed that agonistic, affiliative and interspecific social behaviours varied significantly between the periods (Q = 15.416; *p* = 0.0004; Q = 13.535; *p* = 0.0012; Q = 23.444; *p* < 0.0001, respectively). Based on the Nemenyi post hoc test, agonistic behaviours were performed more in the late-reunion period than in the post-reunion periods A and B (Nemenyi test: *p* = 0.023; *p* = 0.040, respectively). Affiliative behaviours were observed more in the late-reunion period than in the post-reunion period A (Nemenyi test: *p* = 0.006). Finally, interspecific social behaviours were observed more in the late-reunion period than in the post-reunion periods A and B (Nemenyi test: *p* < 0.0001; *p* = 0.045, respectively). No significant differences emerged between the post-reunion periods A and B (Nemenyi test: *p* = 0.127).

##### Update to Present

From 11 September 2017 (56 days after reunion) to 13 October 2017 (88 days after reunion), the cats were involved in a placebo-controlled experimental study on the effects of F3 used as a form of sensory enrichment. The first 5 days of such an experiment, being in the baseline phase, were included in the present paper as the late-reunion period. During the study, there was no significant difference in the duration of agonistic behaviour among the phases. Agonistic behaviours were performed less than affiliative ones (1.6% vs. 4.6% on the total observation time, respectively) (data not shown, Bertoni et al., in prep.). One-hundred and one days after reunion, the non-reintroduced youngest female was found dead in the enclosure; ten days later, the oldest female cat (the mother) was found dead in the enclosure. In both cases, necropsy findings were compatible with them having been attacked and killed. This case embodies why there is a need for more knowledge on the procedures used and the outcomes of (re)union attempts in zoological institutions. We can only speculate in very general terms on the events that occurred. A possible hypothesis is that the cats’ group failed to stabilise again after the reunion, despite all the precautions taken to minimise the risk of such an event. Another hypothesis is that, as the European wildcat is a solitary animal with very little overlapping among the territories of (even related) females [51], the intolerance among the study’s adult cats was independent of the fact that RF was temporarily removed from the group and could have happened even if she had always remained in the enclosure with other cats. This case embodies why there is a need for more knowledge on the procedures used and the outcomes of (re)union attempts and, in more general terms, on the possible harms and benefits of keeping animals of solitary species in group/pairs in zoological gardens.

On 8 March 2018, the cats were relocated to a new, bigger enclosure and released simultaneously into the new environment. The novelty of the area was a distraction and a stimulation for the wildcats, discouraging social tensions. No other noteworthy events occurred after the release.

## 4. Discussion

Given the scarcity of scientific evidence and published reports on the outcomes of (re)union attempts in zoological institutions for European wildcats, we aimed at investigating the reunion process of a female of this species in her family group. As in the domestic cat, the introduction of new individuals in the previous six months can be linked with a higher occurrence of agonistic behaviour [52]. We used the CAP semiochemical to facilitate social interactions between the wildcats and to reduce the likelihood of aggressive interactions. In the present study, agonistic behaviours were almost absent in the post-reunion period immediately after the reunion, even being significantly lower than in the pre-reunion period. They were shown again in the late-reunion period, in which they were significantly more expressed than in the post-reunion periods A and B. In the laboratory, in short-haired domestic cats treated with CAP spot-on, the remanence of the semiochemical is 28 days from its provision (Cozzi, Pers. Comm.). Thus, as the late-reunion period started 56 days after the studied wildcats were spotted with the CAP, this increase might be because the semiochemical’s appeasing effect was no longer present. The trend was the same for intraspecific affiliative social behaviours, which were displayed significantly more during the late-reunion period than in the post-reunion period A. Affiliative behaviours are recognised as being linked to positive emotional states, and thus, improved welfare [53,54,55]; in all periods, intraspecific affiliative behaviours were more expressed than agonistic ones.

After the reunion, the observed wildcats also performed significantly less attentive behaviour and self-grooming than in the pre-reunion period. In zoo animals, vigilance behaviours have been described as an adverse effect of zoo visitor presence [56,57,58,59]. Self-grooming is a typical behaviour of felids that involves cleaning the fur and the control of ectoparasites [60]. However, the reduction in self-directed behaviours in zoo big cats, such as lions, suggests positive welfare implications [61]. Indeed, self-directed behaviours, such as self-grooming, when overperformed, could signal a stressful or anxious situation, underlining a compromised state of well-being [62,63,64,65].

One behaviour that could have suggested a possible negative effect of the procedure was the increase in time spent being unobserved in the post-reunion period compared to the pre-reunion period. This behaviour was shown in particular by one of the study subjects (the sister of RF). However, the time spent out of sight (hiding or staying away from the visitor/observer area) can be informative of both the need to be elusive and to cope with a stressful situation in wild animals in zoos [8,56,57,58,66,67,68,69,70]. However, in such a case, an increase in alertness and vigilance would have been expected, whereas there was a decrease in attentive behaviour after reunion. Attention increased again in the late-reunion period and was significantly higher than in the post-reunion period A. Exploration also increased over the study periods. In particular, exploration was performed significantly less in the post-reunion period A than in both the post-reunion period B and in the late-reunion period. An increase in exploration, an active species-specific behaviour, is interpreted as a sign of an increased welfare level in wild felids under controlled conditions [53,61,71,72,73,74].

Although group stability was not attained in the long term, in the first days after the reunion, the animals overall did not show signs suggesting that they were overly stressed and even appeared to have an elevated aggression threshold. In this regard, it is important to note that we cannot draw any definite conclusions about the role of CAP itself independently of the other aspects of the applied reunion procedure based on the changes found in the wildcats’ behaviour. Notwithstanding, it is noteworthy to mention that CAP administration has been found to have a positive effect on social behaviour [35,36] and decrease the agonistic behaviour of domestic cats and to be beneficial in the reunion of new individuals with a social group in Sumatran tigers [2]. Contrary to the former species who show behavioural plasticity in their choice of social vs. solitary lifestyle depending on resources [75], the latter is known to be a highly socially intolerant species [76]. As the European wildcat is also a solitary species, in which adults disperse within one year of age and females tend to have non or minimally overlapped territories [51], one hypothesis could be that pheromones (CAP) were able to mitigate the stress of the reunion for a period after their remanence, with similar positive effects to those found in tigers.

After that, the aggression threshold may have begun to reduce gradually, leading to very severe overt aggression and killing after several months. If this was the case, pheromones can be a useful means to open a window of opportunity for the cats to coexist pacifically, in which the staff should implement dedicated behavioural interventions aimed at promoting and ensuring group stability in the long term, even after pheromone withdrawal.

On the other hand, it must be borne in mind that the studied cats, as it often occurs in zoological gardens [77], were kept as a group—a situation that differs from what is observed in the wild for this solitary species. Thus, random episodes of intolerance among adult solitary animals could occur independently of the fact that one of them had been temporarily removed.

In any case, future long-term research involving a larger sample of wildcats and a greater number of species of felids hosted in a zoo is needed to better understand the functioning and effects of semiochemicals in the ex situ management of these species to promote choices that safeguard the welfare of the animals in a controlled environment as much as possible.

## 5. Conclusions

The present paper is, to our knowledge, the first study that documents the behavioural responses of a group of wildcats to the reunion of one member of the social group after a long time of separation due to veterinary reasons. The reunion procedure was gradual, and CAP was used on all of the cats to minimise agonistic behaviour and to increase acceptance. In the absence of other published literature about (re)unions in this species, it is somewhat difficult to understand the outcome of the described reunion procedure. On the one hand, the behaviour of the cats after the reunion seemed to suggest that there were no problems in the group. On the other hand, there were tragic attacks among the cats three months after the reunion. Therefore, as (re)unions occur in zoological settings and are very challenging situations for most animal species, with this paper, we aim to break the ice and strongly advocate the need for systematic publications on the strategies enacted and on the outcomes of (re)union episodes in facilities housing animals worldwide to contribute to the formulation of evidence-based best practices on the subject.

## Figures and Tables

**Table 1:** All the study periods are summarised. Ten days of data collection were conducted for both the pre-reunion period and the post-reunion period (PR1-PR10; POR1-POR10, respectively). The post-reunion period was divided into two periods: post-reunion period A (POR1-POR5) and post-reunion period B (POR6-POR10). Five days of data collection were run for the late reunion (LR1-LR5). R1 indicates the beginning of the reunion process, in which we provided semiochemicals to all individuals and released RF and the mother; R2 indicates the second day, when all five wildcats were together again.

PR1	PR2	PR3	PR4	PR5	PR6	PR7	PR8	PR9	PR10	R1	R2	POR1	POR2	POR3	POR4	POR5	POR6		POR7	POR8	POR9	POR10	Pause from 29/07 to 10/09	LR1	LR2	LR3	LR4	LR5
Pre-reunion period										Reunion period		Post-reunion period											Pause 44 days	Late-reunion period				
										Spot-on	All cats together	Post-reunion period A						Post-reunion period B										

**Table 2:** Ethogram of the study.

Behavioural Categories	Definition
Individual behaviours	
Attention	A wildcat is alert and stares at a specific point with straight ears or with ears backwards.
Maintenance	A wildcat defecates and then covers faeces, urinates, eats and drinks. Includes stretching, body shake, individual play, manipulation of plants and other objects and yawning after waking up.
Exploration	A wildcat explores the environment visually or olfactorily (sniffing the ground or any object).
Self-grooming	A wildcat cleans its fur by licking, scratching, biting, chewing or by licking a paw and swiping it on the head with the apparent intent to clean the head.
Locomotion	A wildcat walks, runs or jumps inside the enclosure.
Territorial behaviours	A wildcat marks the environment by urine spray with vertical tail and horizontal urine jet, clawing, rubbing the head against an object, defecating without covering the faeces and patrolling.
Social behaviours	
Affiliative	A wildcat observes, sniffs or licks another subject or rubs the head and nose against the body of another wildcat.
Agonistic	A wildcat stares at another subject, with ears forward of the head. It can move the tail with fast movements or can have ears flat. Includes agonistic displays, such as hissing, piloerection, raised paw and bared teeth.
Social interspecific	A wildcat observes zookeepers, visitors or animals belonging to species other than its own.
Inactivity	
Individual inactivity	A wildcat sleeps or rests alone.
Social inactivity	A wildcat sleeps or rests in contact with another subject.
Not observed	A wildcat is hiding or is not distinctly visible.

**Table 3:** Behavioural categories performed by wildcats during the pre-reunion and the post-reunion period. For each behavioural category, the table reports the median (IQR) duration in seconds. Moreover, *V* and *p* values of the Wilcoxon test from the comparison between the two periods are also reported. Asterisks indicate significant difference (*p* < 0.05). # Not enough data.

Behavioural Categories	Pre-Reunion	Post-Reunion	*V*-Value	*p*-Value
Individual behaviours				
Attention	8 (72.5)	0 (19)	3.268	0.001 *
Maintenance	6 (18.75)	0 (13.25)	1.347	0.178
Exploration	104.5 (399.75)	12.5 (339.25)	1.320	0.187
Self-grooming	29 (128)	0 (64.25)	2.605	0.009 *
Locomotion	0 (33.75)	0 (22.75)	−0.076	0.939
Territorial behaviours	0 (2.25)	0 (2.25)	−0.033	0.974
Inactivity				
Individual inactivity	696.5 (1060.25)	492.5 (1178)	0.787	0.431
Social inactivity	#	#	#	#
Not observed	0 (4.5)	0 (126.75)	−2.651	0.008 *

**Table 4:** Behavioural categories performed by wildcats across the periods. For each behavioural category, the table reports the median (IQR) duration in seconds. Moreover, *p* values of the Nemenyi test from the comparison between the periods are reported. Asterisks indicate a significant difference (*p* < 0.05).

	Periods		Post-Reunion Period A vs. Post-Reunion Period B	Post-Reunion Period A vs. Late-Reunion Period		Post-Reunion Period B vs. Late-Reunion Period
Behavioural Categories	Post-Reunion Period A	Post-Reunion Period B	Late-Reunion Period	*p*-Value	*p*-Value	*p*-Value
Individual behaviours						
Attention	0 (10.75)	7.5 (45.25)	29.5 (94.5)	0.343	0.002 *	0.114
Maintenance	0 (6.25)	3 (18.5)	7.5 (14.5)	0.270	0.073	0.793
Exploration	9.5 (186.5)	97(439.75)	205.5 (438)	0.011 *	0.001 *	0.793
Self-grooming	0 (15.5)	0 (61)	6.5 (57.25)	0.793	0.248	0.609
Locomotion	0 (4.25)	0 (70.25)	5.5 (89.25)	0.189	0.082	0.916
Territorial behaviour	0 (0)	0 (9.5)	0 (6)	0.871	0.995	0.916
Inactivity						
Individual inactivity	1026 (1108.25)	129.5 (1155.5)	235 (972.5)	0.065	0.127	0.952
Social inactivity	0 (0)	0 (0)	0 (0)	0.988	0.641	0.734
Not observed	0 (79.25)	12 (124.25)	0 (66.75)	0.454	0.952	0.293

## Data Availability

The data presented in this study are available on request from the corresponding author.
